# Preferential sacral fracture sites in fragility fractures of the pelvis type IVb and comparison of internal fixation methods: CT-based morphological mapping and finite element analysis

**DOI:** 10.1007/s00068-026-03087-7

**Published:** 2026-02-27

**Authors:** Shuichi Naniwa, Masanori Yorimitsu, Tsubasa Hasegawa, Teruhiko Ando, Ryuichiro Okuda, Shiro Fukuoka, Yusuke Mochizuki, Yasuaki Yamakawa, Ryuichi Nakahara, Shiro Hanakawa, Toshifumi Ozaki

**Affiliations:** 1 Department of Orthopaedic Surgery, Section of Medicine, Division of Medicine, Dentistry and Pharmaceutical Sciences, Graduate School of Medicine, Dentistry and Pharmaceutical Sciences, Okayama University, 2-5-1 Shikata-cho, Kita-ku, Okayama city, 700-8558 Okayama Japan; 2 Department of Musculoskeletal Traumatology, Faculty of Medicine, Dentistry and Pharmaceutical Sciences, Okayama University, 2-5-1 Shikata-cho, Kita-ku, Okayama city, 700-8558 Okayama Japan; 3 Department of Orthopaedic Surgery, Kagawa Prefectural Central Hospital, 1-2-1 Asahi-machi, Takamatsu city, 760-8557 Kagawa Japan; 4 Department of Emergency Health Care and Disaster Medicine, Faculty of Medicine, Dentistry and Pharmaceutical Sciences, Okayama University, 2-5-1 Shikata-cho, Kita- ku, Okayama city, 700-8558 Okayama Japan; 5 Department of Orthopaedic Surgery, Kochi Health Sciences Center, 2125-1 Ike, Kochi city, 781-0111 Japan; 6 Department of Musculoskeletal Health Promotion, Faculty of Medicine, Dentistry and Pharmaceutical Sciences, Okayama University, 2-5-1 Shikata-cho, Kita-ku, Okayama city, 700-8558 Okayama Japan; 7 Department of Orthopaedic Surgery, Okayama Saidaiji Hospital, 1-1-70 Kanaokahigashi-machi, Higashi-ku, Okayama city, 704-8194 Okayama Japan; 8 Department of Orthopedic Surgery, Faculty of Medicine, Dentistry and Pharmaceutical Sciences, Okayama University, 2-5-1 Shikata-cho, Kita-ku, Okayama city, 700-8558 Okayama Japan

**Keywords:** Fragility fractures of the pelvis, Spinopelvic dissociation, Finite element analysis, Internal fixation

## Abstract

**Purpose:**

Fragility fractures of the pelvis (FFP) classified as Rommens-Hoffman type IVb are associated with spinopelvic dissociation and are generally considered to require surgical intervention. This study aimed to clarify the localization patterns of FFP type IVb and compare the biomechanical stability of different internal fixation techniques.

**Methods:**

In this retrospective study, morphologic mapping of sacral fracture lines was performed in 36 patients with FFP type IVb. Based on the mapping results, a finite element (FE) model of FFP type IVb was developed to evaluate the biomechanical stability of ilio-sacral screw (ISS) fixation, trans-sacral screw (TSS) fixation, spinopelvic fixation (SPF; On each side, L5 pedicle screw was connected to two iliac screws with a rod, and the bilateral constructs were linked using a cross-connector.), and bilateral triangular fixation (one TSS at S1 combined with SPF mentioned above) using finite element analysis (FEA).

**Results:**

Morphologic mapping showed that the sacrum fracture transverse line tended to pass between the S1-2 transverse lines. Although bilateral triangular fixation and SPF provided the highest stability in both U-type and H-type fractures, a TSS for U-type and two TSSs for H-type also demonstrated comparable levels of stability. ISS-based methods showed greater displacements.

**Conclusion:**

TSS-based fixation may provide stability comparable to bilateral triangular fixation and SPF in FFP type IVb, with less invasiveness when anatomy permits. Further studies are needed to optimize treatment strategies for this complex injury.

## Introduction

Fragility fractures of the pelvis (FFP) are becoming increasingly common due to the extension of life expectancy in the elderly population and are often associated with severe pain, reduced mobility, diminished quality of life, and increased mortality rates [1, 2]. This trend is particularly evident in unstable fractures classified by Rommens-Hoffman [3] as type III or IV, which involve displaced fracture of the posterior pelvic ring. Surgical treatment has been considered for such cases, as it has been reported to facilitate early mobilization, alleviate pain, and reduce mortality rates [4–6]. However, the quality of evidence supporting these benefits is still low and no gold standard surgical procedure has been established. Moreover, due to the risks associated with surgical treatment for FFP, including neurovascular injury, implant failure, and perioperative complications specific to elderly patients, both benefits and risks must be carefully considered [6–9].

Despite these limitations, there is a general consensus that FFP type IVb, characterized by spinopelvic dissociation, warrant surgical intervention due to the high degree of instability. A Computed Tomography (CT) scan is highly recommended for diagnosing this fracture type and planning surgery [10]. However, even CT scans have been reported to have a sensitivity of 66.1% for sacral insufficiency fractures [11]. Therefore, it is important to understand the characteristics of the sacral fracture line in type IVb fractures.

Although ilio-sacral screw (ISS) fixation is relatively simple and widely used to minimize the surgical invasiveness, its stability at osteoporotic fracture sites may be insufficient, and screw loosening was frequently reported [12]. Trans-sacral screw (TSS) fixation can transfer vertical loads across the sacrum via a bicortical anchorage, and provide good functional outcomes. However, loosening or reoperation following percutaneous screw fixation has been reported [13], which raises questions about whether sufficient stability can be achieved. Spinopelvic fixation (SPF) bridges the lumbar spine and the ilium with pedicle and iliac screws connected by rods which provides strong construct stability, but stress concentration on the extraosseous rods [14] and potential complications [15] were reported.

Biomechanical studies employing finite element analysis (FEA) are valuable in assessing the mechanical stability of fracture fixation methods. Although several studies have been conducted using FEA models of the pelvis [14, 16, 17], only a limited number of studies have specifically focused on FFP [18].

**Table 1:** Model properties of materials

	Young's modulus (MPa)	Poisson's ratio	Critical tensile stress (MPa)	Yield stress (MPa)	Stress relaxation factor	Crushing strain (μ)
Bone (Heterogeneous materials)	upper limit: 49033.3	0.4	σt=0.8σm	Noelasticity	0.05	10000
	lower limit: 14.7					
Intervertebral disc	5	0.45	5	1	0.1	10000
Interpubic disc	5	0.45	5	1	0.1	10000
Titanium screw	108854	0.28	899	824	0.1	100000
Co-Cr bar	240000	0.28	1450	1000	0.1	100000

Therefore, this study aimed to systematically map the sacral fracture patterns in patients with FFP classified as Rommens-Hoffman type IVb based on CT images for improving the accurate evaluation of posterior component fractures. We also aimed to evaluate initial construct stability across different types of fixation methods using FEA, thereby contributing to the development of optimal management strategies for FFP.

## Materials and methods

### Patient cohort

A retrospective study was conducted on FFP Rommens-Hoffman type IVb cases, treated at our hospital or affiliated institutions between January 2014 and December 2024. Cases with sacralization, lumbarization, other dysplastic anomalies, prior lumbopelvic instrumentation or prior sacral surgery, pathological fractures secondary to tumor, infection, or metabolic bone disease other than osteoporosis, or lack of imaging suitable for three-dimensional (3D) CT reconstruction were excluded. After exclusions, 3D sacral CT images of 36 patients were generated from Digital Imaging and Communications in Medicine (DICOM) data of CT scans. The cohort consisted of 5 males and 31 females, with mean age of 82.9 ± 8.75 years. This study was approved by the Ethics Committee of our hospital (Approval ID; 2502-005, Approval date; 7th May 2025).

### Radiographic evaluation and statistical analysis

Fracture types were classified by their morphology in the coronal plane and were categorized as H-, U-, or T-type [19], classification of Dennis et al. [20], and the location of transverse fracture line.

### Morphologic mapping of the fracture line

A 3D reconstructed image of the pelvis was created from the DICOM data using SYNAPSE VINCENT (Fujifilm Medical Co., Ltd., Tokyo, Japan). The pelvis was slightly tilted backward and the fracture line was evaluated as a sacral frontal view. Fracture lines were traced using the GNU Image Manipulation Program (version 2.10; Spencer Kimball, Peter Mattis and the GIMP Development Team. Available from: https://www.gimp.org). Anterior sacral views were overlaid onto the two-dimensional template created from healthy individual for the tracings, aligning with the first sacral foramina to ensure proper alignment. All figures including this mapping result were created using Inkscape software (version 1.3.2; Inkscape Project. Available from: https://www.Inkscape.org).

### Finite element (FE) model construction

**Fig. 1:**
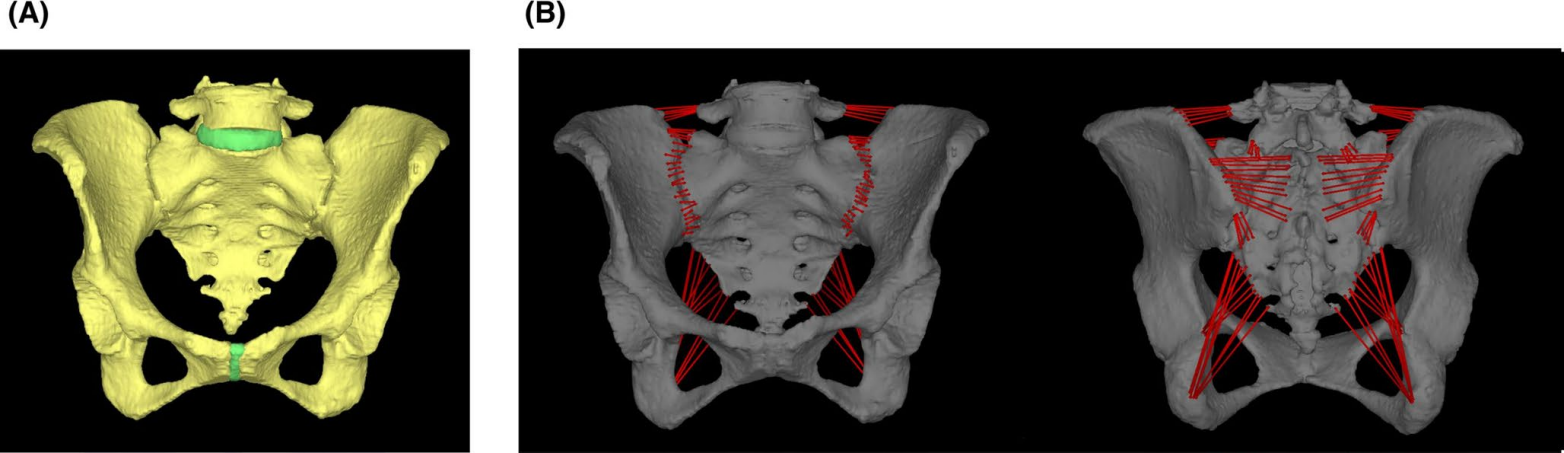
Construction of finite element models. (**A**) The validation model. (**B**) Each ligament was attached to the bone as a truss element (tension-only material)

The FE model was constructed by importing DICOM data of a 70-year-old Japanese female with a hip osteoarthritis into MECHANICAL FINDER (version 13; Research Center of Computational Mechanics, Inc., Tokyo, Japan). The cortical and cancellous bone properties were modeled as heterogeneous materials based on CT-derived bone density values and the software was used for all subsequent analyses. The meshing parameters were set as follows: for bones, the base mesh size was 3 mm with a permissible shape error of 0.2 mm; for intervertebral and interpubic discs, the base mesh size was 3 mm with a permissible shape error of 0.2 mm; and for implants, the base mesh size was 2 mm with a permissible shape error of 0.2 mm. The ligaments included the iliolumbar, anterior sacroiliac, sacrospinous, posterior sacroiliac, sacrotuberous, and joint capsule ligaments, which were modeled as truss elements (tension-only material) attached to the bone (Fig. 1). The implant models were constructed using freeCAD (version 0.21.2; The FreeCAD Community. Available from: https://www.freecad.org). Bone material properties were defined as heterogeneous material based on the CT values as described in a previous study [21], and other materials as shown below (Table 1). The coefficient of friction between bone fragments was set to 0.1 and between a bone fragment to an implant was set to 0.3. The number of elements was 1,194,870 and the number of nodes was 1,003,296 for the validation model without fractures.

### Model validation

As part of validation, previously reported studies have shown displacements ranging from 1.33 to 1.61 mm in FE models [22] and 0.973–1.550 mm in cadaveric pelvises [23] under a vertical load of 500 N applied to the superior surface of L5. Under the same loading condition, the displacement observed in our model was 1.58 mm. For validation of fixation methods, previous reports using two TSSs under the same loading conditions demonstrated displacements of 1.33–1.69 mm [22, 24] and the displacement of our model was 1.65 mm.

### FEA

**Fig. 2:**
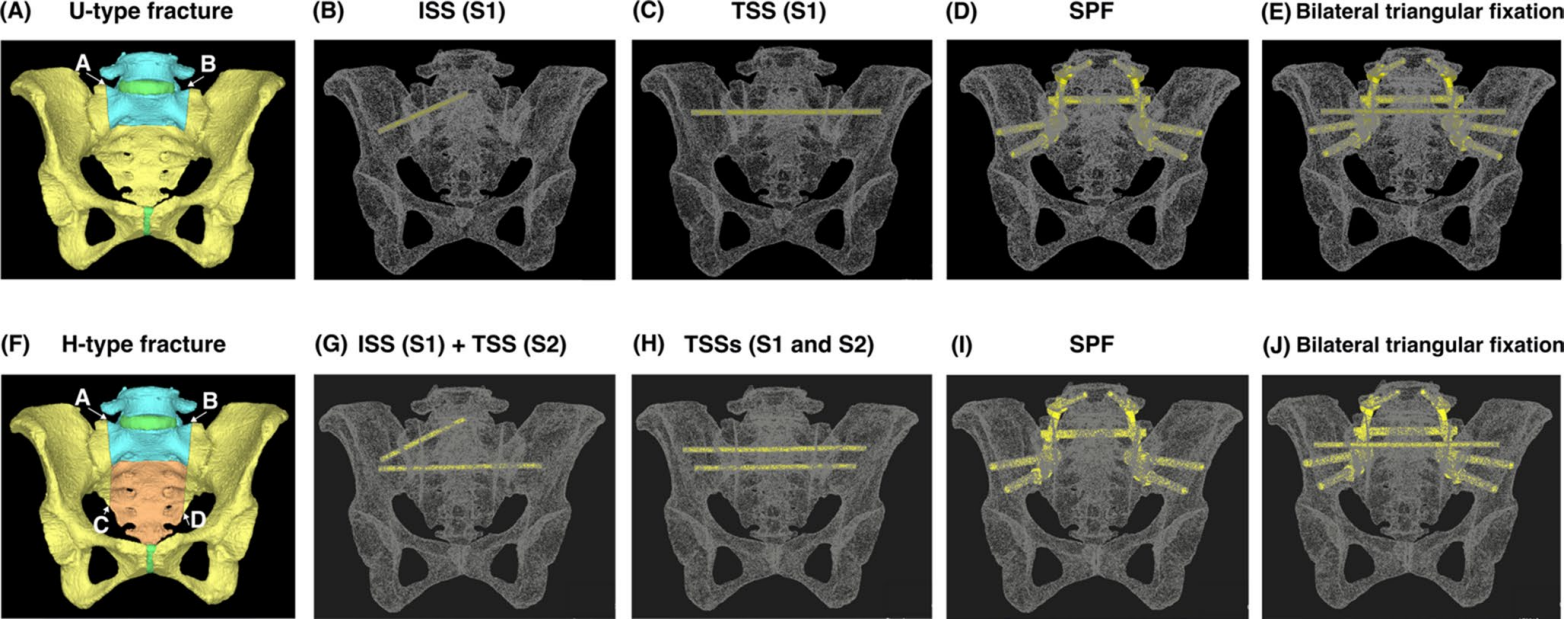
(**A**) The measurement points of displacement for the U-type fracture. (**B**) The image of implant construction of the Ilio-sacral screw (ISS) fixation for U-type. (**C**) The Trans-sacral screw (TSS) fixation for U-type. (**D**) The spinopelvic fixation (SPF) for U-type. (**E**) The bilateral triangular fixation for U-type. (**F**) The measurement points of displacement for the H-type fracture. (**G**) The ISS combined with the TSS fixation for H-type. (H) The two TSSs fixation for H-type. (**I**) The SPF for H-type. **(J**) The bilateral triangular fixation for H-type

FEA is a well-established method that discretizes complex anatomical structures into small finite elements and calculates their mechanical response under defined loading and boundary conditions to quantify stress and strain, and compare fixation mechanics under controlled loading conditions [25]. FE models were created based on the mapping result, to simulate U- and H-type fractures, characterized by the transverse fracture line passing between S1 and S2. For these models, the left hip joint was fixed and a 1000 N vertical load was applied to the superior surface of L5. For the U-type fracture, the 3D displacement at the right upper edge (A) and the left upper edge (B) of the sacral alar fracture were evaluated (Fig. 2A) with a single ISS inserted from the right side at S1 (Fig. 2B), a single TSS at S1 (Fig. 2C), an SPF (Fig. 2D; On each side, an L5 pedicle screw was connected to two iliac screws with a rod, and the bilateral constructs were linked using a cross-connector.), and a bilateral triangular fixation (Fig. 2E; one TSS at S1 combined with SPF mentioned above). For the H-type fracture, the 3D displacement at the right upper edge (A), the left upper edge (B), the right lower edge (C) and the left lower edge (D) of the sacral alar fracture were evaluated (Fig. 2F) with one ISS inserted from the right side at S1 and one TSS at S2 (Fig. 2G), two TSSs at S1 and S2 (Fig. 2H), the SPF mentioned above (Fig. 2I), and the bilateral triangular fixation mentioned above (Fig. 2J). The lateral bend was assessed by measuring the angular change of the right iliac wing between non-loaded and loaded states. The TSS and ISS were made of titanium alloy screws (6.5 mm in diameter), the SPF Iliac screws were made of titanium alloy screws (8.5 mm in diameter), the pedicle screws were titanium alloy screws (6.5 mm in diameter) and the connecting bars for SPF were Co-Cr alloy bars (5.5 mm in diameter).

### Graphical presentation

All graph creation was conducted using GraphPad Prism 10 (GraphPad Software, San Diego, CA, USA).

## Results

### Fracture mapping of FFP type IVb showed that the transverse fracture line most commonly passed between the S1-2 transverse line

**Fig. 3:**
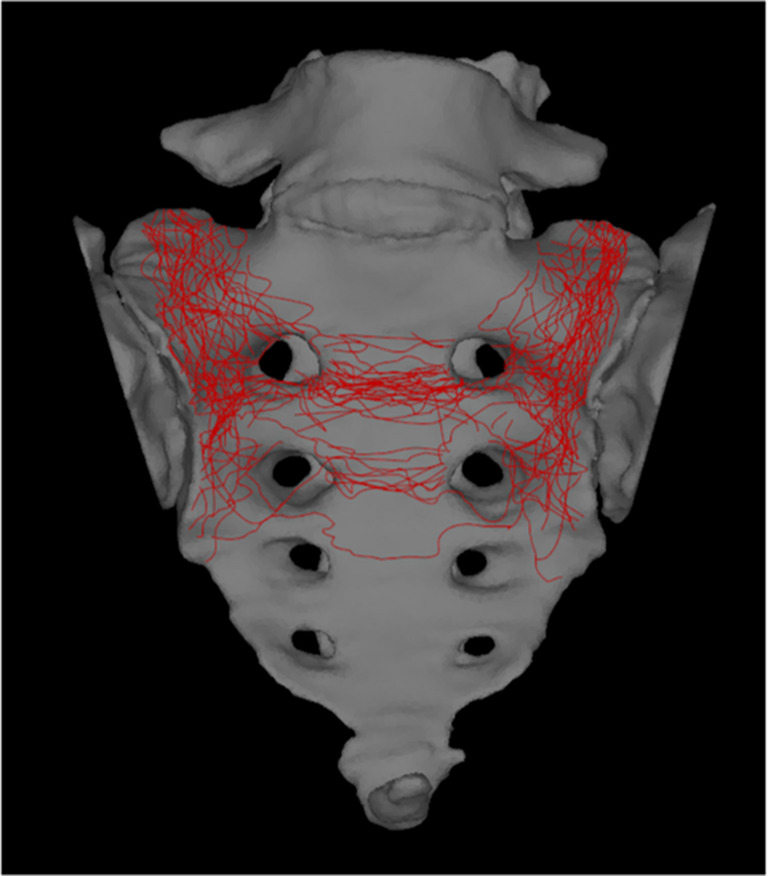
The morphologic mapping of sacral fractures of FFP type IVb showed that the vertical line passed through zone 1 in all cases

The morphologic mapping showed that 16 patients were the U-type, 15 were the H-type, 5 were the T-type, and 0 was the lambda-type. The location of the fracture lines tended to converge within a specific region. Vertical lines passed through zone 1 in all cases. Transverse lines were present between S1 and S2 in 26 cases, between S2 and S3 in 8 cases, between S3 and S4 in 1 case, and between S1, S2, and S3 in 1 case (Fig. 3; Table 2).

**Table 2:** Patterns of fracture types and transverse line distribution

Type of fracture	cases, n (%)
U-type	16 (44.4)
H-type	15 (41.7)
T-type	5 (13.9)
Transverse line	cases, n (%)
S1-2	26 (72.2)
S2-3	8 (22.2)
S3-4	1 (2.8)
S1-2 & S2-3	1 (2.8)

### Comparison of displacements and lateral bends for various fixation methods in FFP type IVb

**Fig. 4:**
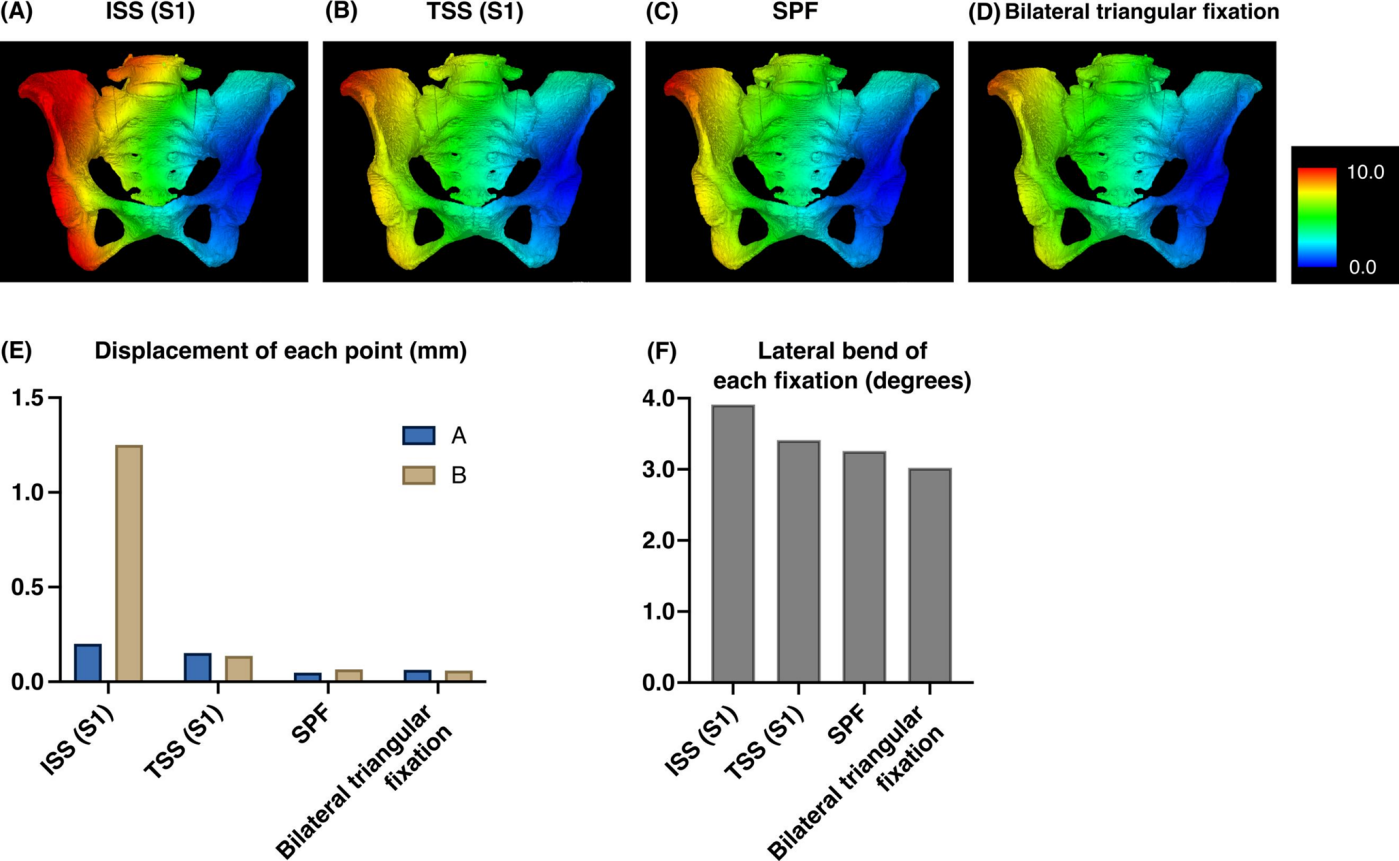
The FEA results for each fixation method for the U-type fracture. (**A**) ISS at S1. (**B**) TSS at S1. (**C**) SPF. (D) Bilateral triangular fixation. The color bar indicates the amount of displacement (mm). (**E**) Displacement of each point with each fixation technique in U-type fractures. (**F**) Lateral bend of each fixation technique in U-type fractures

For U-type fractures, the displacement value under the loading condition was 0.201 mm (A) and 1.251 mm (B) for the ISS at S1, 0.152 mm (A), and 0.137 mm (B) for the TSS at S1, 0.048 mm (A) and 0.066 mm (B) for the SPF, and 0.063 mm (A) and 0.059 mm (B) for the bilateral triangular fixation. Lateral bend was 1.15° for the validation model, 3.91° for the ISS at S1, 3.41° for the TSS at S1, 3.26° for the SPF, and 3.02° for the bilateral triangular fixation (Fig. 4).

**Fig. 5:**
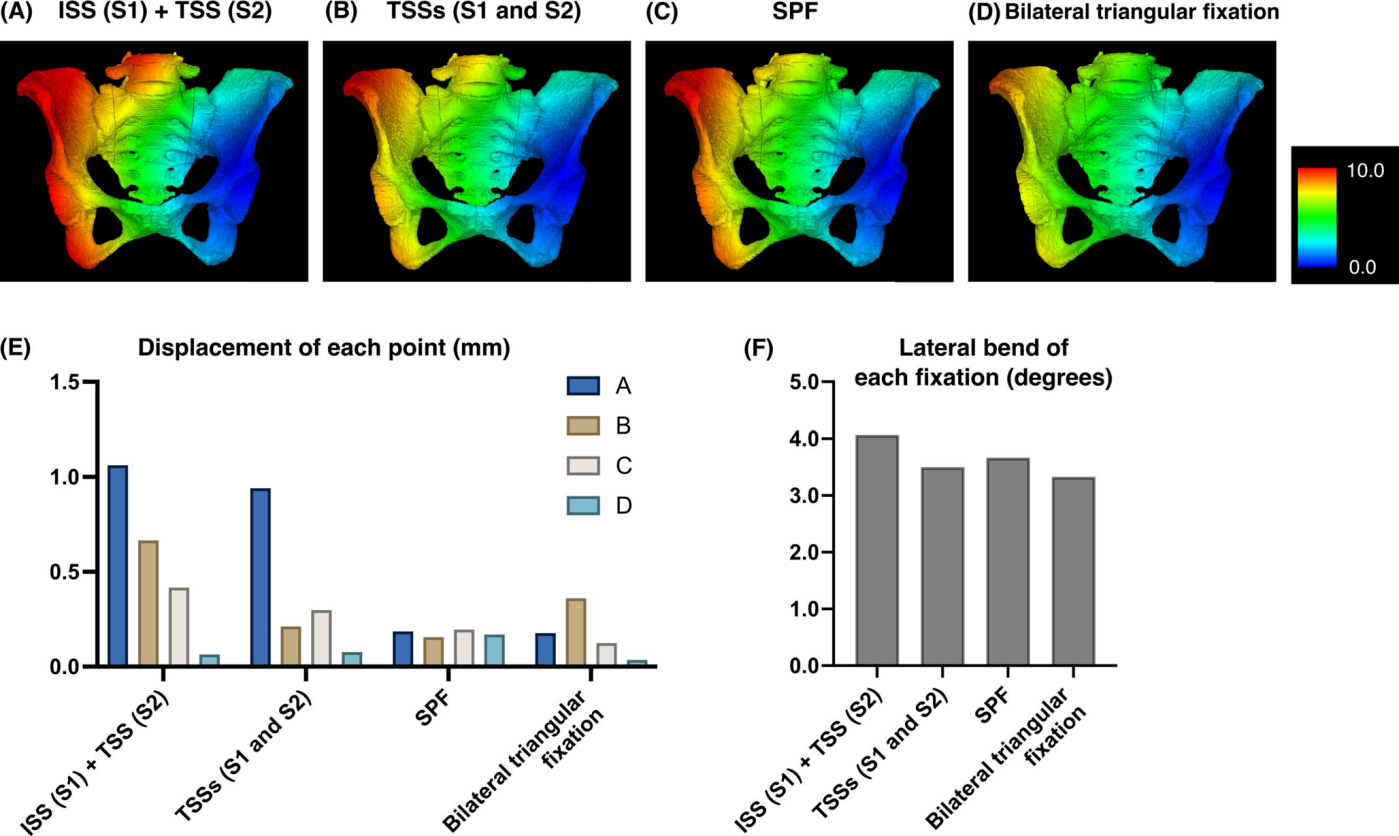
The FEA results for each fixation method for the H-type fracture. (**A**) ISS at S1 + TSS at S2. (**B**) TSSs at S1 and 2. (**C**) SPF. (**D**) Bilateral triangular fixation. The color bar indicates the amount of displacement (mm). (**E**) Displacement of each point with each fixation technique in H-type fractures. (**F**) Lateral bend of each fixation technique in H-type fractures

For H-type fractures, the displacement value under the loading condition was 1.062 mm (A), 0.666 mm (B), 0.417 mm (C), and 0.064 mm (D) for the ISS at S1 combined with the TSS at S2, 0.941 mm (A), 0.212 mm (B), 0.298 mm (C), and 0.077 mm (D) for two TSSs at S1 and 2, 0.185 mm (A), 0.155 mm (B), 0.195 mm (C), and 0.169 mm (D) for the SPF, and 0.117 mm (A), 0.361 mm (B), 0.124 mm (C), and 0.035 mm (D) for the bilateral triangular fixation. The lateral bend was 4.07° for the ISS at S1 combined with the TSS at S2, 3.50° for two TSSs at S1 and 2, 3.67° for the SPF, and 3.33° for the bilateral triangular fixation (Fig. 5).

## Discussion

In this study, we report for the first time that the morphologic mapping of sacral fractures with FFP Rommens-Hoffman classification type IVb revealed a consistent pattern in fracture line distribution. There have been few reports on the fracture sites, other than Rommens et al. [3], that transverse fracture lines tend to occur in S1 or S2. Regarding vertical lines, previous studies have evaluated bone mass CT values of the sacrum [26] and the cortical bone thickness and bone density of the cadaveric sacrum was evaluated from CT values of the sacrum [27]. Furthermore, although the characteristic H-shaped uptake pattern on bone scintigraphy is a well-known imaging feature of sacral fragility fractures [28], no prior studies have addressed the specific location of transverse fracture lines. The concentration of transverse fracture lines observed in this study corresponds to regions referred to as transverse anterior crest [29] or transverse lines. Magnetic resonance imaging (MRI) is useful for accurate evaluation of the posterior component. However, the cost and limited accessibility may restrict the use of MRI in clinical practice. Because a CT scan is highly recommended for this fracture type as the basis for preoperative planning [10], these findings are clinically useful in estimating posterior component fractures, which may still be missed even on CT [11, 30].

Using DICOM data from a 70-year-old female pelvis, we created FE models that reflected the heterogeneous cortical and cancellous bone properties of osteoporotic regions. Validation of displacement was performed under conditions consistent with previous studies, which primarily fixed ischia and applied vertical loads of 500 N. However, in our analysis to simulate a single-leg stance during walking, the loading conditions were modified to fix one hip joint and apply loads of 1000 N. This setup aligns with conditions used in prior biomechanical studies involving cadaveric pelvises [31]. Our findings showed that in U-type fractures, the amount of displacement was smallest and comparable between bilateral triangular fixation and SPF, followed by TSS, although those fixations resulted in minimal displacements of less than 0.2 mm. In contrast, ISS demonstrated displacement exceeding 1 mm on the non-instrumented side. Lateral bend was smallest with bilateral triangular fixation, followed by SPF and then TSS. In H-type fractures, bilateral triangular fixation, SPF, and two TSSs appeared to provide greater stability as none of those fixation methods resulted in displacements exceeding 1 mm. However, due to the anatomic characteristics of the S1 corridor [32], TSS placement at S1 is not always feasible, so ISS at S1 for U-type and a combination of ISS at S1 and TSS at S2 for H-type are frequently selected in clinical settings. A report based on FEA indicated that these fixation methods combining ISS and TSS provided superior stability compared to ISS fixation alone [33]. Nevertheless, these methods demonstrated the largest displacement in our analysis and screw loosening has been frequently reported [12, 13]. Although stability of SPF is robust, multiple recent studies comparing SPF and trans-sacral fixation have reported equivalent postoperative clinical outcomes for both techniques [34–36]. As a single TSS for U-type and two TSSs for H-type also demonstrated fixation strength comparable to bilateral triangular fixation and SPF in the current study, and considering its less invasive nature, TSS may be considered the preferred method when S1 corridor is anatomically available.

In determining the optimal fixation strategy for FFP, the following factors should also be taken into consideration.

Among patients with FFP type IVb, some exhibit acute U-type or H-type sacral fractures caused by a single trauma event, however, others show a transition from FFP type I or II to progressive instability with incomplete healing and fragile callus formation [37, 38]. In these cases, the final fracture configuration may not exhibit the same degree of instability typically observed in classic spinopelvic dissociation. For cases of acute U-type or H-type, isolated fixation with ISS or TSS may be biomechanically insufficient, highlighting the potential need for more comprehensive stabilization strategies. From our results, bilateral triangular fixation and SPF appeared to provide similarly high stability, the use of bilateral triangular fixation may be excessive in this context. To establish definitive fixation methods in FFP type IVb, further research is required. This includes developing more accurate FE models, conducting biomechanical studies using cadaveric pelvises, and performing prospective clinical studies stratified by the interval between initial injury and the completion of type IVb fracture patterns.

This study had several limitations. First, the FE model was created from DICOM data from a single Asian patient and therefore should not be generalizable to the broader population. Second, vertebrae cephalad of L4 and femurs were excluded and soft tissues that were not constructed were not included. The mechanical behavior observed here may differ from that of the whole body. In addition, FEA is entirely virtual, and its reliance on simplifications also creates weaknesses. These weaknesses make it prone to misunderstandings and errors in implementation and interpretation. Third, each generic fracture line was inserted linearly and dynamic analysis was not performed, so the data may not correspond to the actual fracture behavior. Fourth, FEA results are relative and useful for comparison between fixation methods but absolute evaluation of dislocation and lateral bend requires other empirical investigations. Fifth, in vivo fracture healing is a complex, time dependent, and biologically regulated process that cannot be completely captured by a static structural model. Similarly, because the screw–bone interfaces were idealized without progressive loosening, the analysis may underestimate micromotion and failure mechanisms in osteoporotic bone. We therefore focused on initial construct stability across fixation methods, a key determinant of early micromotion and healing potential. Despite these limitations, in the context of the growing demand for less invasive procedures with adequate fixation strength as alternatives to SPF for FFP type IVb, a particular strength of the current study is that we were able to provide FEA results that may mechanistically support and corroborate the outcomes reported in recent clinical series. Taken together, the findings of this study provide important data and clinically useful insights into the field of FFP, which continues to require further research.

## Conclusion

This study investigated the fracture patterns and biomechanical behavior of FFP type IVb. The morphologic mapping of the fracture lines revealed the tendency for sacral fractures to traverse primarily between the S1-2 transverse line. FEA demonstrated that bilateral triangular fixation and SPF provided the highest stability in both U-type and H-type fracture models, with minimal displacement. However, due to their surgical invasiveness, their routine application in FFP may be excessive. A single TSS for U-type and two TSSs for H-type offered comparable stability to bilateral triangular fixation and SPF, suggesting that TSS may be considered the preferred method when S1 corridor is anatomically available. ISS for U-type and ISS at S1 combined with TSS at S2 could be selected as an alternative method, however, this method showed the largest displacement in the current study. To optimize fixation strategies for FFP type IVb, further research is required. This includes the development of higher-precision FE models, cadaveric studies, and prospective clinical trials.

## Data Availability

No datasets were generated or analysed during the current study.
